# Small energy benefits of in-wake flying in long-duration migratory flights

**DOI:** 10.1098/rspb.2024.1173

**Published:** 2024-09-11

**Authors:** Elisa Perinot, Ortal Mizrahy Rewald, Johannes Fritz, Marco Salvatore Nobile, Alexei Vyssotski, Thomas Ruf, Leonida Fusani, Bernhard Voelkl

**Affiliations:** ^1^ Department of Interdisciplinary Life Sciences, University of Veterinary Medicine, Savoyenstrasse 1a, Vienna 1160, Austria; ^2^ Waldrappteam Research and Conservation, Schulgasse 28, Mutters 6162, Austria; ^3^ Department of Behavioural and Cognitive Biology, University of Vienna, Djerassiplatz 1, Vienna 1030, Austria; ^4^ Department of Environmental Sciences, Informatics and Statistics, Ca’ Foscari University of Venice, Venice, Italy; ^5^ Bicocca Bioinformatics, Biostatistics and Bioimaging Research Center (B4), University Milano Bicocca, Monza, Italy; ^6^ Institute of Neuroinformatics, University of Zurich and Swiss Federal Institute of technology (ETH), Zurich, Switzerland; ^7^ Animal Welfare Division, Vetsuisse Faculty, University of Bern, Laenggassstrasse 120, Bern CH-3012, Switzerland

**Keywords:** formation flight, energy saving, dynamic body acceleration, heart rate, flight strategies

## Abstract

During long-distance migrations, some bird species make use of in-wake flying, which should allow them to profit from the upwash produced by another bird. While indirect evidence supports energy saving as the primary benefit of in-wake flying, measurements are still missing. We equipped migrating northern bald ibises (*Geronticus eremita*) with high-precision global navigation satellite system data loggers to track their position in the flock. We estimated birds’ energy expenditure through different proxies, namely dynamic body acceleration (DBA), heart rate and effective wingbeat frequency. During active flapping flight, DBA estimates dropped off when in-wake compared with when not-in-wake. In addition, effective wingbeat frequency decreased, suggesting an increased use of intermittent gliding flight during in-wake periods. Heart rate varied greatly among individuals, with a clear decrease during gliding—corroborating its energy-saving function. Furthermore, we found consistent proof for decreased heart rate during in-wake flying, by up to 4.2%. Hence, we have shown that flying in the wake of another individual reduces birds’ DBA, heart rate and effective wingbeat frequency, which could reflect reduced energy requirement.

## 1. Introduction

Worldwide, many bird populations perform long seasonal migrations in response to ecological factors [1]. As the energetic costs of migration are high, migrating birds develop multiple strategies to save energy, such as thermal soaring, intermittent flight or travelling in close structured groups (formations) [2]. When a bird is flying, the air flows around the wings and then detaches behind them as wingtip vortices, producing two regions of upwash outboard of the wings and a region of downwash inboard [3,4]. Another bird can exploit the upwash, obtaining free lift and experiencing less drag, which would allow it to save energy [5,6]. In this position, the bird is said to be ‘in-wake’. The first theoretical studies on formation flight suggested that aerodynamic interactions in a flock might enable birds to reduce energy expenditure by more than 50% [4,7]. Yet these studies modelled formation flight using the fixed-wing aerodynamic theory of airplanes, without considering wing dynamics, flexibility and morphology [8].

Empirical data on formation flight are scarce as it is particularly challenging to collect datasets about the precise position of the birds in a flock, especially when coupled with energy expenditure estimates. In a study by Weimerskirch *et al.* [9], the authors trained a small group of white pelicans *Pelecanus onocrotalus* to fly in formation behind a boat and equipped the birds with heart rate loggers. They reported that pelicans flying staggered behind a leading bird showed a reduction in heart rate of up to 14.5% and suggested that the birds were exploiting the upwash produced by preceding birds. In a study with migrating northern bald ibises *Geronticus eremita*, the authors discovered that the birds flying in formation coordinated by flapping spatially in phase, and suggested that it could enable upwash capture and maximize aerodynamic advantage [10]. In another study, Friman *et al*. [11] measured the CO_2_ produced by European starlings *Sturnus vulgaris* while flying in formation in a wind tunnel and reported a 25% decrease in flight costs for in-wake birds. Given the limited amount of empirical data on this behaviour, there is still substantial uncertainty about how much energy birds can save by flying in the wake of another bird, especially during migratory conditions.

In this study, we investigated the hypothesis that formation flight allows birds to reduce energy consumption during migratory flights, and we explored the mechanism by which it is achieved. We postulated that northern bald ibises save energy when they can exploit the upwash produced by a preceding bird compared with when flying solo or at the head of the flock. For this purpose, we equipped 29 birds of a flock of northern bald ibis with data loggers with high-precision global navigation satellite system (GNSS) receivers and accelerometers along six migratory flights of a human-guided migration. Additionally, some birds carried heart rate loggers. The navigation data allowed us to determine the position of the birds in space with centimetre-level accuracy and to calculate the in-wake state of each bird at every time point. To quantify energy savings, we used well-established proxies of energy expenditure (i.e. heart rate [12–15] and acceleration data), from which we extracted dynamic body acceleration (DBA) [16–19] and effective wingbeat frequency. We predicted that (i) both DBA and heart rate would decrease when flying in-wake, and (ii) birds would save energy when in-wake by skipping wingbeats and engaging in short glides.

## 2. Methods

### (a) Experimental design

The data were collected in 2019 within the framework of a European LIFE reintroduction project (LIFE+12-BIO_AT_000143), led by Waldrappteam Conservation and Research (https://www.waldrappteam.at/en/). Thirty-two northern bald ibis *G. eremita* chicks were collected from a breeding colony at Zoo Rosegg in Carinthia, Austria, and hand-raised by two human-foster parents. During summer, the birds were trained to follow two motorized microlight planes with the foster parents as co-pilots. The human-guided migration started in the mid of August from Heiligenberg (South Germany) and ended in the wintering site WWF Oasi Laguna di Orbetello in southern Tuscany (Italy) [20]. The migration totalled 720 km and comprised seven daily flight stages within 12 days from 14 to 26 August. Data collection took place during four of these stages and included also the last two training flights (see electronic supplementary material, table S2). We collected satellite data at 5 Hz for 27–30 birds using custom-made high-resolution GNSS receivers (RTK Consultants LLC, USA). Additionally, all the loggers contained three-dimensional accelerometers. Finally, we equipped four birds with ECG loggers (see electronic supplementary material for technical details). Logger weight did not exceed 38.2 g, which was about 3% of the body mass of the lightest bird [21]. To process the GNSS data, we used the software RTKlib (v. demo5 b33b) [22,23] and Python (v. 3.9.7—main libraries *pandas, numpy* [24,25]). Output consisted in the precise positioning (cm-level accuracy) of the birds for each time stamp in three dimensions (yielded as north|south, east|west and up|down axes, but for simplicity referred in the text as back/forward, left/right and up/down, respectively) and expressed in meters distance (details on post-processing: electronic supplementary material, S1.2).

### (b) Heart rate and acceleration measurement

Details about collection of these data are described in Mizrahy-Rewald *et al.* [26]. Briefly, we recorded heart rate at a sampling frequency of 1600 Hz using the external ECG logger Neurologger 2A (Evolocus LLC, USA) [27] and three adhesive mini electrodes (NeoLead, Connect Medizintechnik GmbH, Austria) fixed to the birds’ skin. For each flight, we equipped four birds with the heart rate loggers, selecting among the heaviest birds in the flock. In several cases, the birds removed the electrodes before departure by pulling on the cables; despite this, we successfully recorded ECG five times. Three axial acceleration between ±2 g was acquired at 20 Hz with an accelerometer (model LIS3DH, STMicroelectronics, Switzerland) integrated in the GNSS logger board.

### (c) Data analysis

We eliminated about 10 min after departure and before landing as the birds took some time to start following the microlight. Moreover, we eliminated timepoints with lower accuracy—tens of centimetres—to reduce noise in the data (mean across flights 1.77%). Finally, we excluded timepoints when birds were using thermals (variable across flights—range 0–30%). We interpolated missing values but only for gaps under 1 s. To calculate the relative position of each bird with every other individual in the flock, we considered every combination of dyads of bird and calculated their relative position with respect to the flight direction [28]. Based on the tri-axal acceleration data, we calculated the vectorial DBA [18,29]. Moreover, using the *z*-axis acceleration (heave), we determined for each time point if the bird was flapping or gliding (electronic supplementary material, S1.3). We calculated heart rate in beats per minute (bpm) from the raw ECG data using the algorithm described by Mizrahy-Rewald *et al.* [26] and Ruf *et al*. [30].

### (d) Birds’ relative position during flight

For each bird and each time point, we calculated the nearest neighbour and the nearest frontal individual in Euclidean distance. For the latter, we distinguished between birds flying co-planar (±0.75 m—half a wingspan) or not. For the nearest frontal individual, we used a kernel density estimation to extract the areas containing 25, 50 and 75% of the observed relative positions.

### (e) Modelling in-wake flying

We modelled in-wake flying using fuzzy reasoning. We chose this approach because up- and downwash have gradual and not crisp boundaries and fuzzy logic allows to consider such uncertainty [31,32]. We developed a fuzzy inference system (FIS) to model the in-wake areas and determine when an individual is flying in the wake of another one [28] (electronic supplementary material, S1.4). The output of the FIS returned the state (in-wake or not-in-wake; hereafter ‘flying position’) of each individual at each time point as well as the identity of the leading bird. For model computation, we used the Python library Simpful, v. 2.5.0 [33].

### (f) Parameters choice

An FIS requires an initial choice of parameters, which we defined based on existing literature (see electronic supplementary material, table S1). However, as we noticed that the areas of highest density of points did not match the position that we expected birds should keep to exploit the upwash, we explored the data using a second set of parameters for the FIS. We defined as *in-wake* the most populated region according to the frontal nearest neighbour analysis. In the manuscript, we refer as ‘pre-defined’ fuzzy set to the parameters we chose consulting the literature, and as ‘re-defined’ fuzzy set to the second approach (see electronic supplementary material, table S1, and figures S2 and S3).

### (g) Synchronization between position and energy expenditure data

DBA and heart rate were downscaled to meet the GNSS sampling frequency (5 Hz). After visual inspection of the acceleration and GNSS data together, we observed that sometimes these data streams were not synchronized. We excluded from further analysis flights of birds in which the shift between the two datasets was clearly more than 1 s (24 bird flights out of 171). Finally, we excluded gliding phases longer than 5 s, as it generally happened after birds had been soaring or were losing altitude (up to 3% of flying time).

### (h) Calculation of flying bouts

We extracted bouts of in-wake and not-in-wake flying with a minimum duration of 2 s. A bout was defined as period of flight in one of the two flying positions; if in-wake, also behind the same leader. We set a minimum duration to: (i) disregard as in-wake brief encounters of two birds crossing their flying paths; (ii) eliminate noise due to short bouts, as birds are not keeping a stable state; and (iii) reduce eventual noise due to small shifts in time of the datasets. We interpolated gaps in the data of less than 1 s when the bird had the same leader before and after the gap. For the analysis with DBA, we had a total of 85 209 and 77 109 bouts of 2 s or longer for the pre- and re-defined set, respectively (electronic supplementary material, table S3). For the analysis with heart rate, we extracted the bouts and check visually heart rate values to exclude points or bouts where data looked not reliable (see electronic supplementary material, S1.6 for details). We ended up with 2658 and 2304 bouts of 2 s or longer (electronic supplementary material, table S3). To investigate how our selection criterion for the minimal bout length affected the outcome, we reconducted all the analysis with bouts with a minimum length of 5 s (results are reported in the electronic supplementary material).

### (i) Effective wingbeat frequency and flapping frequency

For each flying bout extracted, we calculated effective wingbeat frequency and flapping frequency using the heave axis. Effective wingbeat frequency was defined as the average number of wingbeats per second [34]. We determined peaks in the signal using the function *signal.find_peaks()* of the SciPy library [35]. Flapping frequency, instead, was defined as the dominant frequency in the heave axis for each flying bout. To calculate it, we used the function *signal.periodogram()* of the package SciPy and extracted the highest peak in the power spectral density.

### (j) Statistical analysis

All the statistical analysis were carried out using R (v. 4.1.0) [36]. To estimate the effect of the flying position (in-wake or not) on DBA and heart rate, we used linear mixed models [37]. Considering DBA, the variable had a clear bimodal distribution (electronic supplementary material, figure S7), caused mainly by whether the birds were flapping or gliding. Therefore, we fitted separate models for gliding and flapping. For all the models, we included the flying position as fixed effect, and bird identity, date and bird nested in date as random intercept effects. We included all the theoretically identifiable random slopes, that is the random slope of birds’ flying position within all random effects (manually dummy-coded and centred) [38]. Originally, we considered a maximal model including the parameters for the correlations among random intercepts and slopes. However, as these were not identifiable, we removed them from the model [39]. We tested the effect of flying position using the function *lmer()* of the package lme4, v. 1.1.32 [40], whereas we estimated degrees of freedom and *p*-values based on the Satterthwaite approximation using the package lmerTest (v. 3.1.3) [41]. The models were fitted with restricted likelihood, with default setting or with 10 000 iteration and *bobyqa* as optimizer. Before fitting the models, we inspected the predictor and the response variable. In the models regarding gliding, the response was left-skewed, therefore we log-transformed it. After fitting every model, we checked the assumptions of normality and homogeneous residuals. We observed a deviation from these assumptions in the models regarding flapping (electronic supplementary material, figure S14); however, it did not give rise to concern given the big sample size and the recent findings on these models robustness [42]. We checked model stability by excluding the levels of the random effects one at a time. The analyses revealed that the models were stable, especially for fixed effects. We used the function *bootMer()* of the package lme4 to bootstrap model estimates. We had a total of 1 859 398 and 2 073 551 points for the pre-defined set and the re-defined set, respectively.

For heart rate, for each bout we calculated the mean heart rate and the proportion of flapping flight and used this dataset to assess our hypothesis. The response was right-skewed, so we transformed it (reverse score and square root). We included as fixed effects flying position, flapping proportion(‘flap’) and the duration of the bout as well as all the two-way and the three-way interactions. Continuous variables were scaled to increase results interpretability. Finally, bird identity was included as a random factor, and we included the random slope for flying position to count for among-individual variation. We fitted the model using the same functions as with DBA. We checked for normally distributed and homogeneous residuals (electronic supplementary material, figure S14) and for collinearity (function *vif()*, package car—v. 3.1.2 [43]). All assumptions were approximately met (electronic supplementary material, figure S14). We inspected model stability, which suggested discrete stability. Finally, we calculated confidence intervals of model estimates as previously described. Datasets consisted of 2658 and 2304 data points (i.e. bouts) for the pre- and re-defined sets, respectively. Finally, to determine the effect of flying position on effective wingbeat frequency, we first calculated the mean effective wingbeat frequency for each flying position and then we carried out a one-sided paired *t*‐test (*n* = 60). We chose a one-sided test as we had a directed hypothesis and wanted to test whether the effective wingbeat frequency was higher when not-in-wake than when in-wake. When investigating the effect of in-wake state on flapping frequency, we extracted the mean flapping frequency for each flying position and then performed a two-sided paired test (*n* = 60) as we had no clear hypothesis regarding the direction of this effect. In both analyses, we checked for normality and homogeneity of variances.

## 3. Results

### (a) Migration data

The birds successfully completed the human-guided migration. We logged 733 min of flight (range: 45–180 min per flight), corresponding to 523 km (range: 34–127 km; electronic supplementary material, table S2). After removing the first and last 10 min of each flight, we used 607 min for the analysis. On average, we collected positional data for 96.1% of the birds (range: 93–100%) and acceleration data for 83% of the birds in the flock (range: 71–90%). Missing data were due to malfunctioning loggers or battery drainage. Positioning data quality was excellent, with an average of 99.1% of fix solutions of the flight time (range: 98.3–99.9%). Birds flew at an average speed of 44 km h^−1^ (range: 38–50 km h^−1^). Along the flights, the flock was cohesive, and took up an average volume of 2182 ± 6318 m^3^ (32 ± 36 m × 35 ± 39 m × 10 ± 14 m, width × length × height; electronic supplementary material, table S2). Nearest neighbour distance was on average 2.47 ± 12.53 m (median 1.34 m; electronic supplementary material, figure S4).

### (b) Nearest frontal individual

**Figure 1.**
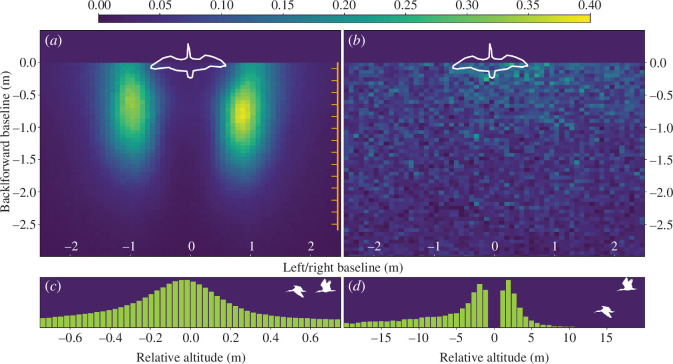
Relative position of the follower relatively to the leader. (*a,b*) Colour-coded density of the follower position relative to the leader (white silhouette) when the two birds are (*a*) flying in one horizontal plane (i.e. with the difference in altitude not exceeding ±0.75 m) or (*b*) at different altitudes (−0.75 m > difference > 0.75 m). Colour bar on top of *a* and *b* is valid for both panels. The orange graduated bar in panel *a* indicates mean chord length for the northern bald ibis. (*c,d*) Distribution of the relative position of the follower relative to the leader on the up/down axis when (*c*) flying in one horizontal plane or (*d*) at different altitudes. Merged data from all birds and all flights; for individual-level data, see electronic supplementary material, figure S6. Graphs’ axes were adapted for illustration purposes.

For each bird (follower), we determined its position relative to the closest bird in front of it (leader). When co-planar (i.e. altitude difference < 0.75 m), followers kept a specific position behind the leaders, hence they preferred flying displaced diagonally (figure 1*a*) but mostly levelled (figure 1*c*). After mirroring positions along the flight axis on the right, we calculated that 25% of the positions were in a volume of 0.34 ± 0.13 m^3^, 50% of the positions in a volume of 1.23 ± 0.39 m^3^ and 75% of the positions were in a volume of 3.86 ± 1.01 m^3^ (see electronic supplementary material, figure S5 and, for all the individuals singularly, electronic supplementary material, figure S6). When not co-planar, followers did not show a preferred position behind the leader (figure 1*b*) but showed a clear preference to fly in a lower position compared to the leader (figure 1*d*).

### (c) Proxies for energy expenditure in the pre-defined in-wake region

**Figure 2.**
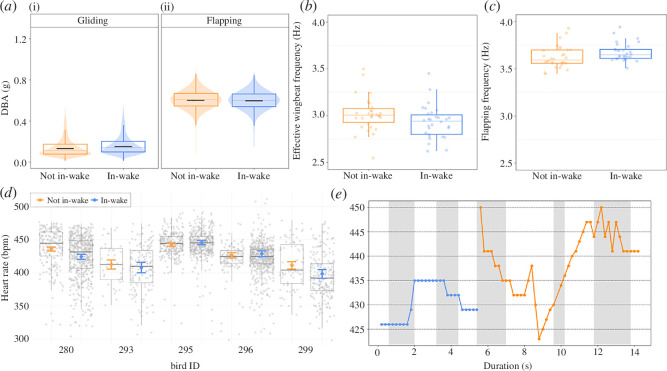
Difference in energy expenditure estimates when in-wake or not-in-wake. (*a*) Boxplots and violinplots showing the difference in DBA estimates determined by flying position during gliding and flapping bouts. The long black line represents the median, whereas the shorter black bar indicates the model fit. Outliers were omitted for illustration purposes. (*b*) Difference in effective wingbeat frequency and (*c*) flapping frequency between the two flying positions. (*d*) Boxplots and scatterplots showing heart rate per bird when in-wake and not-in-wake. The coloured error bars represent the model fit for each bird extracted from the random part of the model. (*e*) An example of heart rate values during an in-wake (blue) and a not-in-wake (orange) bout. Grey background highlights when the bird is gliding during the bout.

Flying position had a significant impact on DBA values. The impact was different depending on whether the bird was gliding or flapping. When gliding, DBA was 16.0% higher in-wake than not-in-wake (*t* = 10.08, d.f. = 6.16, *p *< 0.001; figure 2*a*(i); table 1). When flapping, however, DBA was 1.1% lower when in-wake (*t* = −3.33, d.f. = 4.94, *p* = 0.02; figure 2*a*(ii); table 1) compared with not-in-wake. This difference doubled (~2%) for bouts of 5 s or longer (electronic supplementary material, table S5). Overall, flying position had an impact on heart rate values (full—null model comparison: χ^2^ = 49.8, d.f. = 6, *p *< 0.001), however, it was different across individuals (tables 2 and 3). The analysis of among-individual variation showed that two individuals experienced a decrease in heart rate when in-wake of 2.7 and 3.2%, respectively. For the other three individuals, instead, we could not define the direction of the effect as the intervals crossed zero (table 3; figure 2*d*). For longer bouts, only one individual experienced a decrease of about 3.5% (electronic supplementary material, table S7 and figure S11). Increased flapping caused an increase in heart rate (*t* = −10.12, d.f. = 2524, *p *< 0.001; table 2) of about 2.5% in both flying positions (figure 2*e*).

**Table 1.** Results of the linear mixed model for the pre-defined fuzzy set for DBA. Reported are the estimates, the standard errors (s.e.s), the lower and upper 95% confidence intervals (CIs), *t*-statistic, d.f. based on Satterthwaite approximation and *p*-values.

model	term	estimate	s.e.	lower CI	upper CI	*t*	d.f.	*p*
gliding	intercept^a^	−2.030	0.041	0.121	0.142			
	in-wake^b^	0.147	0.015	0.119	0.177	10.080	6.157	<0.001
flapping	intercept	0.601	0.006	0.589	0.613			
	in-wake^b^	−0.007	0.002	−0.010	−0.003	−3.327	4.944	0.021

^a^ Response was log-transformed.

^b^ Dummy-coded with not-in-wake as reference category.

**Table 2.** Results of the linear mixed model for heart rate, pre-defined set and 2 s bouts. The top table reports the fixed effects, specifically the estimates, s.e.s, lower and upper CI, *t*- and *p*-values, and d.f.

term	estimate	s.e.	lower CI	upper CI	*t*	d.f.	*p*
intercept^a^	9.219	0.346	8.560	9.926	–	–	–
in-wake^b^	0.238	0.203	−0.178	0.659	1.170	4.22	0.304
flap^c^	−0.612	0.060	−0.729	−0.494	−10.124	2524.19	<0.001
duration^d^	0.020	0.033	−0.041	0.086	0.608	2645.92	0.543
in-wake: flap	0.041	0.070	−0.091	0.170	0.581	2485.85	0.561
in-wake: duration	0.051	0.066	−0.174	0.082	0.771	2645.55	0.441
flap: duration	−0.173	0.080	−0.333	−0.009	−2.162	2644.30	0.031
in-wake: flap: duration	0.119	0.109	−0.351	0.084	−1.094	2643.73	0.274

^a^ Due to response transformation, estimates are also transformed (x^2^ – 511.2 where *x* is the estimate to back-transform).

^b^ Dummy-coded with not-in-wake being the reference category.

^c^ Flapping proportion, z-transformed.

^d^ Total duration bout, z-transformed.

**Table 3.** Elaborated results for the random intercepts and slopes for all the individuals for the model in table 2. Italics denote birds for which the direction of the effect is clear.

bird	*280*	293	295	296	*299*
intercept	*8.682*	9.923	8.290	9.207	*9.991*
in-wake estimate	*0.651*	0.229	−0.193	−0.125	*0.628*
in-wake lower interval	*0.441*	−0.156	−0.395	−0.371	*0.316*
in-wake upper interval	*0.860*	0.613	0.009	0.121	*0.940*
% difference	*−2.69*	−1.11	0.72	0.53	*−3.15*
% lower interval	*−3.60*	−3.04	−0.03	−0.53	*−4.78*
% higher interval	*−1.80*	0.74	1.45	1.57	*−1.56*

### (d) Proxies for energy expenditure in the re-defined in-wake region

Flying position had a significant impact on DBA. When gliding, DBA was higher in-wake than not-in-wake (*t* = 10.58, d.f. = 5.37, *p *< 0.001, electronic supplementary material, table S4), which corresponds to a 18.4% increase. When flapping, DBA was lower when in-wake (*t* = −3.06, d.f. = 4.95, *p* = 0.029; electronic supplementary material, table S4 and figure S9 left), corresponding to 1.1% decrease (also in this case, the decrease doubled for bouts of 5 s or longer; electronic supplementary material, table S6). Flying position had also an impact on heart rate values (full–null model comparison: χ^2^ = 34.83, d.f. = 6, *p* < 0.001), however, we observed again great interindividual variability. The analysis of among-individual variation showed that two birds experienced a decrease in heart rate when in-wake of 2.6 and 1.7%, respectively, whereas for other two individuals, heart rate increased (0.8 and 1.1%, respectively). For one bird, instead, we could not define the direction of the effect as the intervals crossed zero (electronic supplementary material, table S8 bottom, figure S12). For longer bouts, two individuals experienced a decrease in heart rate of about 3.8 and 4.2%, respectively (electronic supplementary material, table S9, figure S13). Increased flapping caused an increase in heart rate (*t* = −11.25, d.f. = 2248, *p *< 0.001; electronic supplementary material, table S8) of about 2.7% in both flying positions.

### (e) Effective wingbeat frequency and flapping frequency

From the acceleration on the z-axis (heave), we calculated the effective wingbeat frequency (the average number of wingbeats per unit time) and flapping frequency when birds were in both flying positions according to the pre-defined set of parameters. On one hand, effective wingbeat frequency was on average equal to 3.02 ± 0.19 wingbeat s^−1^ when not-in-wake and 2.93 ± 0.18 wingbeat s^−1^ when in-wake. There was a significant decrease in effective wingbeat frequency when birds were in-wake compared to not-in-wake (*t* = 5.30, d.f. = 29, *p *< 0.001; figure 2*b*), corresponding to a 2.9% drop. At the same time, flapping frequency was slightly higher when in-wake (3.68 ± 0.09 wingbeat s^−1^), than when not-in-wake (3.62 ± 0.12 wingbeat s^−1^). This difference turned out to be significant (*t* = −5.77, d.f. = 29, *p *< 0.001; figure 2*c*), corresponding to a 1.4% increase in flapping frequency. Hence, when birds were not-in-wake the effective wingbeat frequency was 16.6% lower than the flapping frequency, though when they were in-wake the effective wingbeat frequency was 20.4% lower.

## 4. Discussion

In this study, we showed that when flying in the wake of another individual, migrating northern bald ibises experienced a reduction of up to 2% in DBA when flapping and 3% in effective wingbeat frequency. For heart rate, however, we registered a decrease for only two out of five individuals, of up to 4.2%. Considering these measurements and the degree of consistency across different proxies, we see small but valid support for the energy-saving function of in-wake flying during long-distance migratory journeys.

The estimates of our study were modest and fell below our expectations and previously suggested values, especially for heart rate. However, we can identify several key factors that probably explain these results. First, we observed high interindividual variability. Two out of five birds experienced between 1.4 and 4.2 % decrease in heart rate while in-wake, whereas for the other three the direction of the effect was neither clear nor consistent. Yet the number of birds for which we could obtain heart rate measurements was rather limited (five individuals across different flying days) and the datasets across birds were unbalanced, with the flying position not-in-wake being underrepresented. Therefore, we were not able to investigate this variability any further. Second, we expect a certain time delay in heart rate adjustment following changes in activity. Such a carry-over effect might lead to a slight overestimation of heart rate during in-wake flying and a slight underestimation of heart rate when not-in-wake, specifically when flying bouts were short. Indeed, the median bout lengths in our data was 3 and 3.6 s for the pre- and re-defined set, respectively, and less than 19% (33% for the re-defined) of all the bouts lasted 5 s or longer. Another key factor is that the birds in this study were all juveniles during their first migration, probably with little experience on how to fly efficiently in formation. Birds mainly performed short in-wake bouts, which might stress their inability to keep a stable position in the formation. In a study on the ontogeny of formation flight in juvenile *Eudocimus albus*, birds were observed to gradually developed their ability to fly in formation over a period of several months before their first migration, learning from adult individuals [44]. Before the human-guided migration, our birds made extensive training flights; however, they never flew with adult conspecifics, which might be important models for them. Social learning has been shown to be an essential source of information for juvenile birds, for example to learn the migration path [45,46] or to better navigate among thermals [47]. In addition, northern bald ibises do not use line formation extensively but rather use a mix of different strategies including thermal soaring and gliding [34]. As such, this species might show fewer stable formations and reduced ability to profit from formation flight than specialists like certain goose species. Finally, despite our success in improving the measurement of position and in the proxies for energy expenditure, there were limits to the precision of our measurements. Specifically, we reported an occasional offset in the timestream of the acceleration data—although we do not consider this a severe problem given that the offset was always in the sub-second range. Despite everything, our small estimates could also indicate that formation flight under long duration, real-world conditions might deliver only small energy savings for birds, contrary to what has been shown in theoretical studies [4,7], short duration flights [9] or controlled settings [11].

Interestingly, we found evidence of energy savings not only with the theoretical optimal wingtip overlap but also when the overlap was greater. Previous theoretical studies assumed that the optimal lateral distance should be such that the wingtip stays out of the downwash region. Badgerow & Hainsworth [7] and Hainsworth [48] discussed that energy saving was maximized for optimal wingtip overlap and calculated a potential for energy saving of up to 51% compared to solo flight. Nevertheless, energy conservation was still expected, albeit at reduced levels, for greater or minor wingtip overlap values. In line with our results, numerous empirical studies on the position of the birds inside the formation reported a high variability in wingtip spacing [6,9,10,48], suggesting that birds might struggle to keep a stable position [49]. Alternatively, we propose that: (i) the main upwash region might primarily support the bird’s body instead of its wings, leading to different expectations regarding the optimal wingtip overlap; or (ii) inferring the upwash region from the maximum wingspan might lead to an overestimation of the optimal distance, as birds might keep their wings slightly flexed during portions of the flapping cycle.

Contrary to heart rate, DBA during gliding phases was higher in-wake, which would contradict our findings if this were indicative of increased muscular work. However, this increase in DBA might derive from turbulence experienced by the birds, which developed into rotational motion (i.e. roll, pitch and yaw). In fact, when gliding, birds are keeping their wings stretched out and they might be not in the same flapping phase as their predecessor. However, the vortex wake is not straight behind the bird that produces them but undulating, as it follows the movement of the wingtips [50,51]. Therefore, the bird in-wake gliding might enter and exit the wake with its wings—or even the bod—causing the increased DBA estimates. Yet DBA estimates are still low compared with flapping flight, meaning that it does not have a great impact on birds’ body acceleration.

When in-wake, the reduced effective wingbeat frequency was a result of birds skipping wing flaps more often. Specifically, we estimated that birds in-wake were able to skip up to 5.4 wingbeats per minute of flight. Many bird species can use intermittent flapping flight to save energy (i.e. flapping flight interrupted by short gliding phases). Indeed, our results confirm that heart rate increased for higher rate of flapping. A study from our group had already reported that also northern bald ibises can use this flight mode [26]. When in-wake, birds performed intermittent flight at an increased rate, presumably because of the lifting effect of the upwash. Yet, flapping frequency was higher when birds were in-wake, hence followers flapped their wings a bit faster. Although flapping faster leads to an increased energy consumption, it is possible that birds still saved energy thanks to the higher amount of wing flaps skipped. As a matter of fact, this might have played a role in lowering the estimates of energy savings in this study. Interestingly, the effective reduction of wingbeats during in-wake flying that we could observe in this study, is similar to the reduction of 1.7–3.4% reported by Weimerskirch *et al*. for white pelicans [9].

The relative position northern bald ibises kept when flying behind another individual was in line with previous results [10,11,52,53] and shows that in any case, birds clearly avoided the area directly behind the former individual by flying in a laterally staggered formation. This position arrangement has also been observed in several bird species that generally do not fly in line formations, such as different species of shorebirds [54], or cliff swallows flying in tandem [55]. The authors of those studies suggested that the lateral offset could serve many purposes, such as better coordination during flight, collision avoidance or saving energy. Additionally, several explanations have been brought forward for why birds travel in flocks. For example, pigeons can improve navigational accuracy by making use of the wisdom of the crowd [56,57].

Here, we showed that the ibises may profit from flying in the wake of another individual. Thanks to acceleration and high-precision positioning data from all birds of a migrating flock, and heart rate measurements from a subset of individuals collected over several flights, we provided empirical evidence of small but consistent effects, suggesting that in-wake flying works as an energy-saving mechanism during migratory flights. For two individuals, heart rate decreased during in-wake flying, suggesting a reduced metabolic demand. DBA showed a decrease during in-wake flying overall, and specifically during active flapping flight. Finally, effective wingbeat frequency allowed us to estimate that by flying in the wake of another bird, the ibises could increase the number of intermittent glides—a less energy-demanding flight mode. In sum, our work carries the study of formation flight and in-wake flying forward and offers evidence that birds could use it to reduce energy expenditure during migratory flight. Yet real-world, long-duration benefits could be reduced compared with those suggested by theoretical approaches or recordings under more controlled and constrained conditions. Formation flight continues to be challenging to study in natural settings.

## Data Availability

Data and code for this paper can be accessed on Zenodo [58]. Supplementary material is available online [59].
